# Brain development and the attention spectrum

**DOI:** 10.3389/fnhum.2015.00023

**Published:** 2015-02-05

**Authors:** Itai Berger, Anna Remington, Yael Leitner, Alan Leviton

**Affiliations:** ^1^The Neuro-Cognitive Center, Hadassah-Hebrew University Medical CenterJerusalem, Israel; ^2^Department of Psychology and Human Development, Centre for Research in Autism and Education, Institute of Education, University College LondonLondon, UK; ^3^Tel Aviv Sourasky Medical Center, Dana-Dwek Children's Hospital, Tel Aviv UniversityTel Aviv, Israel; ^4^Boston Children's HospitalBoston, MA, USA

**Keywords:** spectrum, attention, ADHD, brain, child development

Decreased attention span, hyperactivity, distractibility, and impulsivity are sensitive but non-specific brain functions and behavioral patterns. These expressions of altered functioning should be acknowledged as non-specific, rather than trying to fit them into existing diagnoses.

These abnormalities should be viewed in terms of underlying developmental processes and not as components of discrete non-overlapping disorders. The tendency to squeeze a group of symptoms into a diagnostic entity has the potential to lead to a non-accurate diagnosis, a less than successful treatment plan, and has the potential to be of little prognostic value (Berger and Nevo, 2011).

The developmental approach can provide predictions as to how characteristics associated with attention change over time, and how multiple risk and protective factors influence these temporal changes. It also has the potential to more readily anticipate associated co-morbid features and disorders (Berger and Nevo, 2011; Visser et al., 2014).

Attention Deficit Hyperactivity Disorder (ADHD) seems to be considerably more common than other diagnoses among “double-diagnoses” given to children with developmental dysfunctions. Yet the exact prevalence, neurobiological mechanisms, genetic and epigenetic modifications, diagnostic difficulties and treatment methods have not been clearly identified or quantified.

During the last years, the number of publications in this field has grown substantially, but, in part, due to the wide range of interested professionals, these studies have been published in a wide range of journals, sometimes missing some of their “target” populations.

In this research topic, we have focused on the latest research on the biological and neural pathways, as well as on psychosocial and behavioral correlates of brain development and attention spectrum. Thirty-Eight contributors representing the broad spectrum of professions involved in clinical and research aspects of attention in 11 articles, including original research, review, mini-review, and opinion articles, provided a broad scope of state-of-the art research in order to enhance our knowledge regarding this new conceptualization of attention as a complicated spectrum.

This research topic challenges the reader to view attention in new conceptual ways, including: focusing on brain maturation delay among otherwise healthy children diagnosed with ADHD compared to their age group (Berger et al., 2013); the effects of age and task load on attention success (Remington et al., 2014); the attentional function among children with fetal alcohol spectrum disorder (Lane et al., 2014); the differential diagnosis of sensory modulation disorder and ADHD (Yochman et al., 2013); the effects of environmental distractors on attentional performance (Cassuto et al., 2013); and the much debated effect of alpha-linolenic acid supplementation on ADHD symptoms (Dubnov-Raz et al., 2014).

This research topic also addresses innovative aspects of attention which are discussed in relation to extreme prematurity (O'Shea et al., 2013), the co-occurrence of ADHD and autism (Leitner, 2014), the limited visual orientation ability of children with autism (Landry and Parker, 2013), the possible effects of sex hormones on attentional abilities (Haimov-Kochman and Berger, 2014), and the possibility of elevating hope among ADHD children through virtual reality (Shiri et al., 2014).

We hope that this topic will provide the reader with exciting and thought provoking aspects about the mechanisms underlying attention, and pointing where this field is headed in terms of developing our understanding of the link between brain development and attention performance.

As such, this research topic seeks to serve as a useful tool for a wide range of professionals with special interest in the unusual aspects of attention in order to increase their knowledge, sensitivity and treatment methods among our patients.

## Conflict of interest statement

The authors declare that the research was conducted in the absence of any commercial or financial relationships that could be construed as a potential conflict of interest.

## References

[B1] Berger I. Nevo Y. (2011). Early developmental cues for diagnosis of attention deficit/hyperactivity disorder in young children. Dev. Disabil. Res. Rev. 17, 170–179. 10.1002/ddrr.1111 23362036

[B2] Berger I. Slobodin O. Aboud M. Melamed J. Cassuto H. (2013). Maturational delay in ADHD: evidence from CPT. Front. Hum. Neurosci. 7:691. 10.3389/fnhum.2013.00691 24298243 PMC3829464

[B3] Cassuto H. Ben-Simon A. Berger I. (2013). Using environmental distractors in the diagnosis of ADHD. Front. Hum. Neurosci. 7:805. 10.3389/fnhum.2013.00805 24319423 PMC3837230

[B4] Dubnov-Raz G. Khouri Z. Wright I. Raz R. Berger I. (2014). The effect of alpha-linolenic acid supplementation on ADHD symptoms in children: a randomized controlled double-blind study. Front. Hum. Neurosci. 8:780. 10.3389/fnhum.2014.00780 25339885 PMC4188038

[B5] Haimov-Kochman R. Berger I. (2014). Cognitive functions of regularly cycling women may differ throughout the month, depending on sex hormone status; a possible explanation to conflicting results of studies of ADHD in females. Front. Hum. Neurosci. 8:191. 10.3389/fnhum.2014.00191 24744721 PMC3978296

[B6] Landry O. Parker A. (2013). A meta-analysis of visual orienting in autism. Front. Hum. Neurosci. 7:833. 10.3389/fnhum.2013.00833 24367314 PMC3856368

[B7] Lane K. A. Stewart J. Fernandes T. Russo N. Enns J. T. Burack J. A. (2014). Complexities in understanding attentional functioning among children with fetal alcohol spectrum disorder. Front. Hum. Neurosci. 8:119. 10.3389/fnhum.2014.00119 24639639 PMC3945927

[B8] Leitner Y. (2014). The co-occurrence of autism and attention deficit hyperactivity disorder in children – what do we know? Front. Hum. Neurosci. 8:268. 10.3389/fnhum.2014.00268 24808851 PMC4010758

[B9] O'Shea T. M. Downey L. C. Kuban K. K. (2013). Extreme prematurity and attention deficit: epidemiology and prevention. Front. Hum. Neurosci. 7:578. 10.3389/fnhum.2013.00578 24065904 PMC3776954

[B10] Remington A. Cartwright-Finch U. Lavie N. (2014). I can see clearly now: the effects of age and perceptual load on inattentional blindness. Front. Hum. Neurosci. 8:229. 10.3389/fnhum.2014.00229 24795596 PMC4005968

[B11] Shiri S. Tenenbaum A. Sapir-Budnero O. Wexler I. D. (2014). Elevating hope among children with attention deficit and hyperactivity disorder through virtual reality. Front. Hum. Neurosci. 8:198. 10.3389/fnhum.2014.00198 24847233 PMC4019862

[B12] Visser S. N. Danielson M. L. Bitsko R. H. Holbrook J. R. Kogan M. D. Ghandour R. M.. (2014). Trends in the parent-report of health care provider-diagnosed and medicated attention-deficit/hyperactivity disorder: United States, 2003-2011. J. Am. Acad. Child. Adolesc. Psychiatry 53, 34–46. 10.1016/j.jaac.2013.09.001 24342384 PMC4473855

[B13] Yochman A. Alon-Beery O. Sribman A. Parush S. (2013). Differential diagnosis of sensory modulation disorder (SMD) and attention deficit hyperactivity disorder (ADHD): participation, sensation, and attention. Front. Hum. Neurosci. 7:862. 10.3389/fnhum.2013.00862 24379772 PMC3863757

